# Sander’s Compound

**Published:** 1859-12

**Authors:** 


### SANDER'S COMPOUND.

Those who attended the Convention at Niagara, and those who read our report of the discussions, recollect that Dr. Forbes introduced to the notice of the Convention a compound for filling teeth, suggested and prepared by Enno Sanders, a chemist of St. Louis. Dr. F. kindly furnished us some of the material, which we have carefully tried, but find it will not answer the purpose. It promises pretty well at the start, but becomes porous, and disintegrates. It is no better than our own experiments, in the same direction, in the spring of 1856, which we did not think worthy of public notice. We are sorry that this, too, is a failure; but we can make nothing else of it. W.

